# Physiological Effects of Ergot Alkaloid and Indole-Diterpene Consumption on Sheep under Hot and Thermoneutral Ambient Temperature Conditions

**DOI:** 10.3390/ani6060037

**Published:** 2016-06-02

**Authors:** Michelle L. E. Henry, Stuart Kemp, Frank R. Dunshea, Brian J. Leury

**Affiliations:** Faculty of Veterinary and Agricultural Sciences, University of Melbourne, Parkville 3010, Australia; stuartkemp@pasturewise.com.au (S.K.); fdunshea@unimelb.edu.au (F.R.D.); brianjl@unimelb.edu.au (B.J.L.)

**Keywords:** alkaloid, perennial ryegrass toxicosis, heat stress, physiology, sheep

## Abstract

**Simple Summary:**

Perennial ryegrass endophyte toxicosis in sheep, caused by ingestion of alkaloids, is characterized by staggers and heat stress resulting in poor production or, in some cases, death. This study investigated effects of alkaloid consumption in sheep housed under thermoneutral versus hot ambient temperature on some production and physiological responses. Alkaloid ingestion and extreme heat produced a similar heat stress response and, when combined, this response was exacerbated. Productivity and health and welfare may be compromised when sheep are consuming alkaloids, and this effect is most severe when alkaloid consumption occurs under hot environmental conditions.

**Abstract:**

A controlled feeding study was undertaken to determine the physiological and production effects of consuming perennial ryegrass alkaloids (fed via seed) under extreme heat in sheep. Twenty-four Merino ewe weaners (6 months; initial BW 30.8 ± 1.0 kg) were selected and the treatment period lasted 21 days following a 14 day acclimatisation period. Two levels of two factors were used. The first factor was alkaloid, fed at a nil (NilAlk) or moderate level (Alk; 80 μg/kg LW ergovaline and 20.5 μg/kg·LW lolitrem B). The second factor was ambient temperature applied at two levels; thermoneutral (TN; constant 21–22 °C) or heat (Heat; 9:00 AM–5:00 PM at 38 °C; 5:00 PM–9:00 AM at 21–22 °C), resulting in four treatments, NilAlk TN, NilAlk Heat, Alk TN and Alk Heat. Alkaloid consumption reduced dry matter intake (*p* = 0.008), and tended to reduce liveweight (*p* = 0.07). Rectal temperature and respiration rate were increased by both alkaloid and heat (*p* < 0.05 for all). Respiration rate increased to severe levels when alkaloid and heat were combined, indicating the short term effects which may be occurring in perennial ryegrass toxicosis (PRGT) areas during severe weather conditions, a novel finding. When alkaloid ingestion and heat were administered separately, similar physiological responses occurred, indicating alkaloid ingestion causes a similar heat stress response to 38 °C heat.

## 1. Introduction

Perennial ryegrass (PRG) (*Lolium perenne*) is the primary forage used in the cool temperate, winter-spring rainfall region of Australia because of its persistence and productivity [1]. This persistence and productivity is largely due to the presence of an endophyte (*Epichloe festucaevar Lolii.*), an internal living fungi which exists in a symbiotic relationship with the plant. The endophyte produces alkaloids during stressful times of the year (mainly summer and autumn) for the plant to reduce insect attack and overgrazing and increase plant persistence and productivity [2]. One important alkaloid, ergovaline, has been found to produce heat stress [3,4,5], reduced prolactin secretion [6,7] and reduces animal production [4,5,8]. The compound lolitrem B causes tremors and staggers which has been well documented in research undertaken in New Zealand [5,9,10,11]. When animals consume infected forage, the condition perennial ryegrass toxicosis (PRGT) can occur. 

Perennial ryegrass toxicosis (PRGT), is a significant animal disease in Australia. During a severe outbreak in 2002, there were 29,000 confirmed sheep deaths, however, it is thought this number only represented a third of farms affected [1]. Body temperatures were observed to rise above 41 °C and respiration rates increased, causing heat stress in sheep [1]. Previous work undertaken under grazing conditions in Australia have shown heat stress (increased respiration rate) when grazing Coopworth ewes on Victorian perennial ryegrass and subterranean clover mix [12]. The cost of PRGT in Australia has been estimated to be $100 million per annum [13].

A previous study undertaken by the authors [14] investigated the interaction between alkaloid consumption and moderate heat stress (maximum ambient temperature 33 °C) and demonstrated a strong interaction between alkaloid consumption and moderate heat conditions. However, southern Australia experiences summer conditions which can reach and exceed 40 °C, far in excess of that studied previously. Furthermore, the concentration of alkaloids in PRG peaks during summer raising the question of the effects of alkaloids in sheep under hot ambient temperature conditions. Research undertaken in New Zealand which has focused on the heat stress effects of PRGT in sheep has focused on grazing studies in natural New Zealand environmental conditions [15]. While this research has found evidence of hyperthermia, these symptoms have only been observed occasionally in the field [15]. Further, summer and autumn ambient temperature conditions in Australia are found to be harsher, as compared to ambient temperature conditions in New Zealand. The aim of this study was to investigate the short-term effects of PRG alkaloids ergovaline and lolitrem B in sheep housed under thermoneutral *vs.* hot ambient temperature, as experienced in summer and autumn in Australia, on some production and physiological responses. 

## 2. Materials and Methods

The experimental protocol was reviewed and approved by the University of Melbourne, Science, Optometry and Vision Sciences and Land and Environment Animal ethics Committee (704727.1). 

### 2.1. Animal Management

Twenty four Merino ewe weaners aged 6 months (30.8 ± 1.0 kg) were sourced from The University of Melbourne (Victoria, Australia), Dookie campus. Sheep were housed in group pens for seven days (initial acclimatisation period), followed by a seven day acclimatisation period to the metabolism crates (steel crates with a wire mesh floor measuring 1.5 m long and 0.6 m wide and raised 0.5 m from the ground) and controlled temperature room before a 21 day treatment period. Sheep were fed a high fibre pellet (9.9 MJ/kg ME and 11.6% CP) with the addition of whole barley (12.8 MJ/kg ME and 12.0% CP), throughout all stages of the experiment, twice daily, ad libitum. Ad libitum feed intake was maintained by increasing feed offered by 10% to sheep which consumed 90% of feed the prior day.

### 2.2. Experimental Protocol

Sheep were PRG naïve at the beginning of the experiment. Alkaloid was administered via whole wild-type endophyte infected PRG seed and this was mixed with whole barley to improve palatability of the seed. Most sheep consumed the mix of seed and barley presented, however, there was some individual sheep variability, with some sheep sorting through the feed, avoiding the seed. The use of this simple mixing method proved to be the most effective way of monitoring and ensuring sheep consumed the seed presented. A previous study using goats found only a small percentage (1.6%) of whole perennial ryegrass seed passes through to the faeces [15] so seed was fed whole. Additionally, previous studies by the authors have found feeding seed whole produces consistent PRGT effects [14,16]. Lolitrem B levels are higher than ergovaline in plant tissue [17], in the current study seed was fed which contained lower levels of lolitrem B compared to ergovaline. This method of alkaloid administration was chosen due to the study focusing on the effects of ergovaline, additionally, fresh pasture or hay was not available for use. Sheep were stratified by liveweight, into groups of four sheep and then within each group randomly assigned to a treatment group. Alkaloid concentration was fed at a Nil level (NilAlk) where sheep received no PRG seed (since, at the time of the experiment, no nil endophyte infected seed was available), and a moderate level where sheep received alkaloid (Alk) via whole infected PRG seed at a concentration of 80 μg/kg LW ergovaline and 20.5 μg/kg LW lolitrem B. The seed fed contained 11.8 ppm lolitrem B and 25.9 ppm ergovaline (not including ergovalinine). This level is very high as compared to levels in pasture which may fluctuate between 1.0–1.6 ppm ergovaline and 2.4–3.9 ppm lolitrem B at peak times in summer and autumn in Australian pastures [18]. Two temperature controlled rooms were used, one was kept at thermoneutral conditions (TN; 16–18 °C) and the other was heated (Heat; 38 °C between 9:00 AM and 4:00 PM and 21–22 °C between 8:00 PM and 9:00 AM).This resulted in four treatment groups, NilAlk TN, NilAlk Heat, Alk TN and Alk Heat. Two groups of six sheep moved through each room. 

Alkaloid dosing levels were based on ergovaline and lolitrem B concentrations used in previous studies [14,16], when moderately high concentrations of alkaloids were fed, resulting in compromised animal production and a heat stress response. However, in these studies sheep did not consume all alkaloid offered, possibly because of feed aversion. Therefore, this experiment aimed at feeding a low to moderate dose of lolitrem B and ergovaline to ensure the consumption of alkaloid. 

Sheep were fed seed/barley at 8:00 AM while pellets were fed twice daily (8:00 AM and 4:00 PM) with the seed/barley mix provided first to ensure consumption. Feed and water intake were measured daily. Alkaloid intake was estimated by measuring the amount of seed in the feed refusal each day. Liveweight was measured on day 0 and 21, prior to feeding. Urine and faecal separators were placed on the metabolism crates on days 5 and 11–17, and 24 h 10% volume/weight samples of faeces were collected and volume of urine was recorded. Faecal samples were weighed and dried at 100 °C for 24 h or until samples reached a stable weight and the dry matter (DM) content was determined. 

Rectal temperature, respiration rate and skin temperature (measured on the back and leg) were measured at three hourly intervals commencing at 8:00 AM and finishing at 5:00 PM on days 2, 5, 9, and 12 of the treatment period. Rectal temperature was measured using a hand held digital thermometer (Vega Technologies Inc., Taiwan, China). Respiration rate was measured by counting the number of breaths taken, via flank movements, in 30 s intervals. Skin temperature was measured on the back and hind leg by parting the wool and holding a hand held digital thermometer (Vega Technologies Inc., Taiwan, China) to the skin for 30 s.

Ambient temperature and relative humidity were measured using a temperature and humidity probe with a data logger attached (DigiTech, New South Wales, Australia). 

### 2.3. Statistical Analysis

Statistical analysis was undertaken using the Genstat statistical package (16th Edition, VSN International, Hemel Hempstead, UK). For all parameters significance was analysed using general analysis of variance. The model included fixed effects of alkaloid (NilAlk *vs.* Alk), temperature (TN *vs.* Heat) and time (hour, day or week depending on the parameter), and the random effect was sheep ID. For DMI, faecal water, urine output, and physiological parameters the data was analysed for weekly responses. To analyse changes over a 9 h period on day 12 for physiological parameters the model included fixed effects of alkaloid, temperature and time (hour), and the random effect was sheep ID. Covariates were also included in the analyses using baseline data or day 0 data where appropriate.

## 3. Results

### 3.1. Environmental Conditions

**Figure 1:**
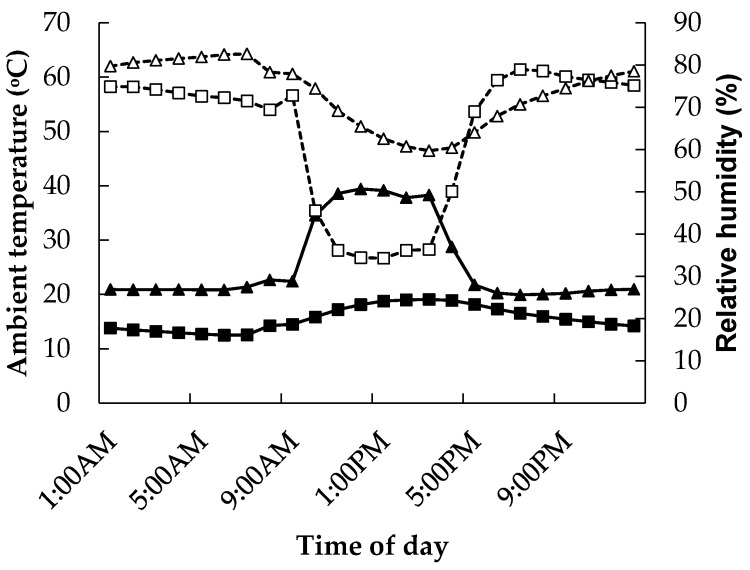
Ambient temperature (▲, ■) and relative humidity (Δ, □) measured under constant thermoneutral temperatures (16–18 °C) (■, □, respectively) and under variable heated conditions (38 °C 9:00 AM–5:00 PM and 21–22 °C 5:00 PM–9:00 AM) (▲, Δ, respectively).

Mean climatic conditions for the study are presented in Figure 1. Ambient temperature remained at TN conditions on day 0 for all treatments. 

During days 1–21 between 9:00 AM and 4:00 PM, in the Heat treatment, the means ± SE for ambient temperature and relative humidity were 38.0 ± 0.11 °C and 29.0% ± 0.22%, respectively. Between 4:00 PM and 9:00 AM, mean ambient temperature and relative humidity for the Heat treatments were 21.4 ± 0.04 °C and 56.7% ± 0.17%, respectively.

### 3.2. Production Response

Sheep consumed 88% of the infected seed offered in the Alk TN treatment and 68% of infected seed in the Alk Heat treatment (*p* ˂ 0.001).

**Figure 2:**
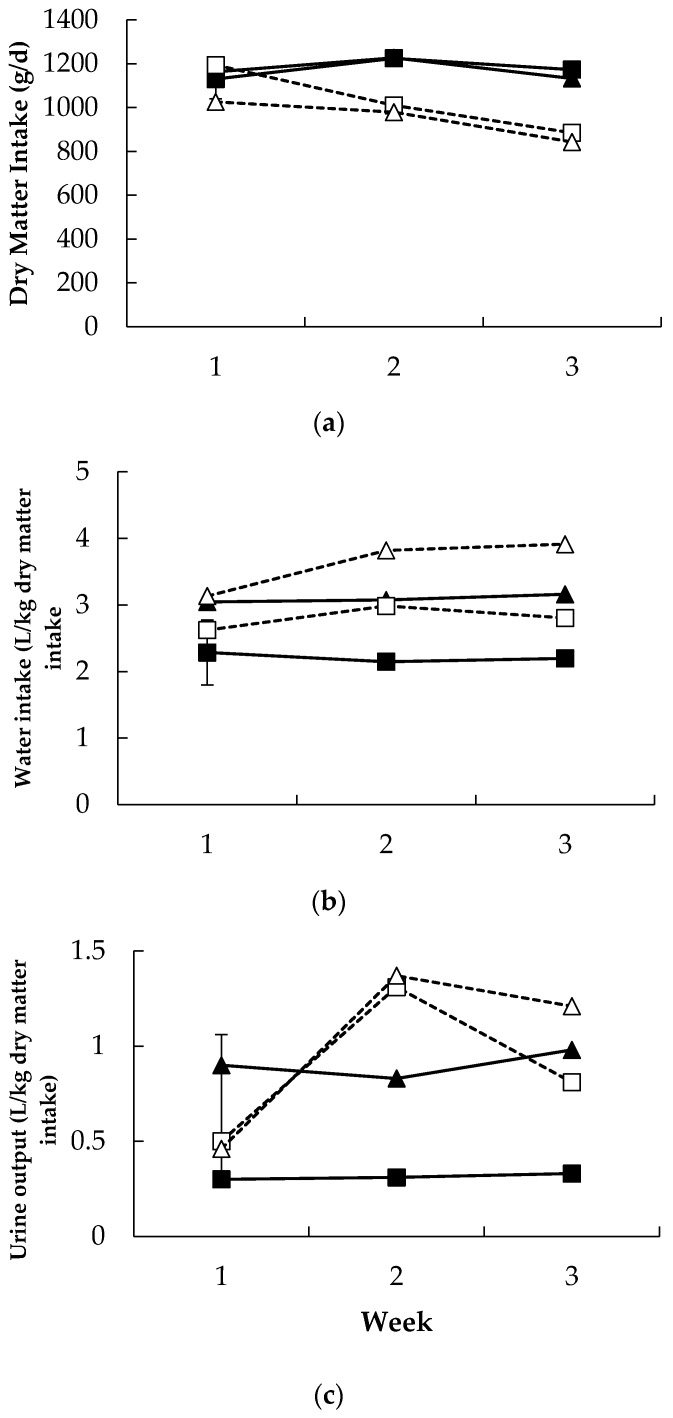
Relationships between (**a**) dry matter intake, (**b**) water intake, and (**c**) urine output and week of study in Merino sheep fed diets containing no (■, ▲) or added perennial ryegrass alkaloids (□, Δ) under either thermoneutral (squares) or heat (triangles) conditions. The *p*-values for the effect of alkaloid, temperature, week and the interaction between week and temperature, week and alkaloid and week, alkaloid and temperature were 0.008, 0.53, <0.001, 0.35, <0.001 and 0.02 for dry matter intake, 0.09, 0.02, 0.02, 0.17, 0.008, 0.68 for water intake and 0.25, 0.39, 0.09, 0.65, 0.04 and 0.87 for urine output, respectively. The standard error of the difference for the three-way interaction is displayed on the data from the sheep receiving no alkaloids under thermoneutral conditions. There were no other significant interactions.

DMI was lower in the Alk treatment compared to the NilAlk treatment (1179 and 1010 g/d; for NilAlk and Alk, respectively; sed 57.1; *p* = 0.008; Figure 2a) but was not altered by temperature (*p* = 0.53). There was a significant alkaloid × week interaction (*p* ˂ 0.001; Figure 2a) such that DMI in the NilAlk treatment remained relatively stable while DMI decreased over time in the Alk treatment. There was an interaction between alkaloid × temperature × week, such that the Alk Heat treatment decreased DMI to the lowest level compared with the other treatments (*p* = 0.02; Figure 2a).

Average daily gain over the experimental period tended to be less for the Alk treatment (*p* = 0.07; Table 1) but was not affected by temperature (*p* = 0.44; Table 1). There was a trend for final liveweight to be lower in the Alk treatment (32.2 and 30.8 kg for NilkAlk and Alk, respectively; sed 1.0; *p* = 0.07), while temperature had no effect (31.8 and 31.2 kg, for TN and Heat, respectively; sed 0.74; *p* = 0.44). There were no significant interactions for liveweight or ADG. 

There was a tendency for water intake to increase in the Alk treatment (*p* = 0.09; Figure 2b), while heat increased water intake over the entire treatment period (*p* = 0.02). There was an interaction between alkaloid and week, such that water intake increased over the treatment period in the Alk treatment (*p* = 0.008). Urine output was variable in the Alk treatment over the experimental period, increasing largely over the first week (*p* = 0.04; Figure 2c). Faecal water and DMD were not altered by alkaloid or temperature (Table 1).

### 3.3. Physiological Response

#### 3.3.1. Overall Experimental Period

Data presented for all parameters has been averaged over all sampling days and times.

Data for rectal temperature and respiration rate are presented in Table 2. Overall, rectal temperature was higher in the Alk treatment (39.73 and 40.13 °C, for NilAlk and Alk, respectively; sed 0.07; *p* < 0.001) and in the Heat treatment (39.80 and 40.07 °C, for TN and Heat, respectively; sed 0.07; *p* = 0.002). There was an alkaloid x week interaction (*p* = 0.001; Table 2) such that rectal temperature decreased over time in the Alk treatment compared with the NilAlk treatment. Overall, respiration rate was higher in the Alk treatment (105 and 153 breaths/min, for NilAlk and Alk, respectively; sed 7.3; *p* < 0.001) and Heat treatment (102 and 156 breaths/min, for TN and Heat, respectively; sed 7.3; *p* < 0.001). There was a tendency for a temperature × week (*p* = 0.07) interaction such that respiration rate increased over time in the Heat treatment but not in the TN treatment. 

Data for skin temperatures are presented in Table 2. Overall back skin temperature was higher in the Alk treatment (38.70 °C and 38.98 °C, for NilAlk and Alk, respectively; sed 0.08; *p* = 0.004) and Heat treatment (38.53 and 39.13 °C, for TN and Heat, respectively; sed 0.08; *p* < 0.001). Similarly, leg skin temperature was higher in the Alk (38.54 and 38.76 °C, for NilAlk and Alk, respectively; sed 0.07; *p* = 0.008) and Heat treatments (38.30 °C and 39.00 °C, for TN and Heat, respectively; sed 0.07; *p* < 0.001). There was a significant alkaloid × week interaction for leg skin temperature (*p* = 0.01; Table 2) such that temperature decreased over time in the Alk treatment but not in the NilAlk treatment. 

#### 3.3.2. Temporal Patterns in Physiological Response

**Figure 3:**
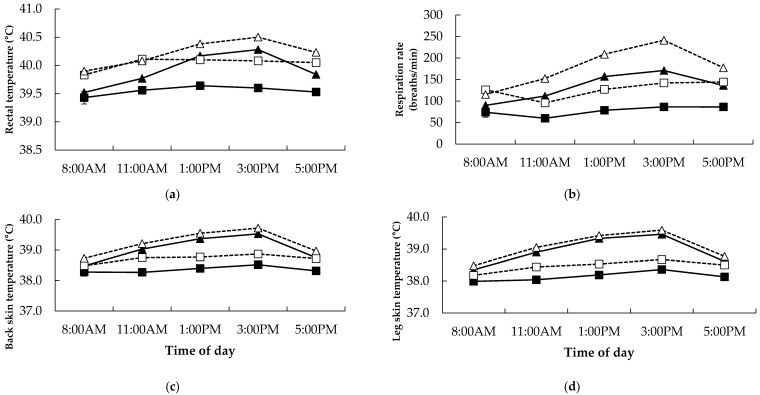
Relationships between (**a**) rectal temperature, (**b**) respiration rate, (**c**) back skin temperature, and (**d**) leg skin temperature and time of day in Merino sheep fed diets containing no (■,▲) or added perennial ryegrass alkaloids (□, Δ) under either thermoneutral (squares) or heat (triangles) conditions on day 12 of treatment. The *p*-values for the effect of alkaloid, temperature and time and the interaction between alkaloid and time, temperature and time and alkaloid and temperature and time were <0.001, 0.002, <0.001, 0.35, <0.001 and 0.33 for rectal temperature, <0.001, <0.001, <0.001, 0.009, <0.001 and 0.044 for respiration rate, 0.004, <0.001, <0.001, 0.82, <0.001 and 0.39 for back skin temperature and 0.008, <0.001, <0.001, 0.75, <0.001 and 0.86 for leg skin temperature, respectively. The standard error of the difference for the three-way interaction is displayed on the data from the sheep receiving no alkaloids under thermoneutral conditions. There were no other significant interactions.

Over the nine hour sampling period on day 12, rectal temperature increased in the Alk and Heat treatments (*p* = 0.008 and *p* ˂ 0.001, respectively; Figure 3a). There was a temperature × time (*p* ˂ 0.001) interaction such that the Heat treatment was variable over time compared to the TN treatment.

Respiration rate increased in the Alk and Heat treatments (*p* ˂ 0.001; Figure 3b). There was an alkaloid × time interaction (*p* = 0.009; Figure 3b) such that the Alk treatment increased respiration rate over time compared to the NilAlk treatment. Further, there was a temperature × time interaction (*p* ˂ 0.001, respectively; Figure 3b) such that the Heat treatment increased respiration rate over time compared the TN treatment. Moreover, respiration rate was higher in the Alk Heat treatment compared to all other treatments at all times points apart from 8:00 AM (*p* ˂ 0.05), while the Alk TN and NilAlk Heat groups were similar at most time points (apart from 8:00 AM and 3:00 PM).

Back and leg skin temperature increased in the Alk and Heat treatments (*p* = 0.004 and *p* ˂ 0.001 for back, Alk and Heat, respectively and *p* = 0.008 and *p* ˂ 0.001 for leg, Alk and Heat, respectively; Figure 3c,d). There were significant temperature × time interactions for back and leg skin temperature (*p* ˂ 0.001) such that the Heat treatment increased skin temperature over time, while the TN treatment remained lower.

## 4. Discussion

### 4.1. Production Response

A key finding from the present study was that DMI was decreased by 22% in response to consumption of rye grass seed that contained moderate levels of endophyte alkaloids. A reduction in DMI due to alkaloid consumption has been reported in previous studies by the same authors when thermoneutral and moderate heat conditions were used for the same period of time [14,16]. Furthermore, this finding is consistent with a previous study which found feed intake decreased in cattle fed endophyte infected tall fescue (containing ergovaline) under 32 °C ambient temperature conditions [3]. Liveweight gain was not significantly affected in this study and this is likely due to the short time period used. Liveweight has been found to decrease in a number of grazing based PRGT studies [19,20,21,22], and with controlled alkaloid intake under thermoneutral and moderate heat conditions [14,16]. A recent review has highlighted the clear production effects of ergot alkaloid consumption [8], reporting the clear link between decreased DMI and liveweight. Further, a recent review has documented clear production effects on sheep consuming perennial ryegrass alkaloids in New Zealand, reporting that liveweight can decrease in sheep, prior to the development of staggers [5]. The findings of the current study support this report.

Faecal moisture increased in the AlkTN treatment in week 1, however, DMD was not reduced by alkaloid or heat in this experiment, contrary to the findings in a previous study [14]. The concentration of lolitrem B consumed in this study was lower than previously used [14] and while increased faecal water has been established previously [15], the alkaloid responsible for increased faecal water has not been clearly identified, lolitrem B has been implicated [23]. This raises the possibility that more marked effects on faecal water and, potentially DMD, may be associated with higher concentrations of lolitrem B intake rather than ergovaline. 

Water intake relative to DMI was increased in response to alkaloid consumption (+14%) and in response to Heat (+15%) with these effects being additive. A previous study measured water intake when sheep were exposed to wild-type and AR37 perennial ryegrass pasture. This study found water intake to increase by 25% when sheep consumed wild-type pasture and when ambient temperature stayed above 20 °C [24] which is consistent with the current study. Further, the increase in water intake due to heat is consistent with the increased heat load in these animals and similar observations in other studies [25,26,27]. The implications of increased water intake could be extremely detrimental to the Australian sheep industry. Urine output relative to DMI was unaffected by alkaloid consumption and did not increase due to heat exposure, although urine output was highly variable and lack of significant treatment effects is perhaps not surprising. The early increase in urine output may have been in response to the initial consumption of alkaloids, and a result of altered electrolyte and fluid balance [28]. The subsequent decrease in urine output may have been due to the decrease in DMI and therefore total alkaloid consumption. Similarly, the decrease in alkaloid consumption and short period of time used were the likely causes of little change in liveweight, even though DMI decreased significantly.

### 4.2. Physiological Response

Alkaloid ingestion and heat resulted in increased rectal temperature and respiration rate. This is probably due to the relatively high heat load imposed by the heating regimen. Presumably, the magnitude of this was such that elevation of respiration rate was insufficient for heat to be dissipated adequately during the TN period as indicated by the 9 h physiological profiles presented in Figure 3b. Respiration rate was extremely high in the Alk Heat treatment during the heat period, well above the other treatment groups. This finding indicates the severity of additive effects of alkaloid and heat, and begins to explain the severity of heat stress which may be occurring during summer in southern Australian PRG areas, a novel finding. The mechanism responsible for these increases may be due to the binding of ergovaline to serotonin-2 receptors causing vasoconstriction [29], resulting in increased rectal temperature and therefore respiration rate in an attempt to reduce heat load. Respiration rate was similar in the NilAlk Heat and Alk TN treatment, suggesting that alkaloid ingestion increased heat load similarly to 38 °C heat. Grazing studies have found increases in rectal temperature and respiration rate over summer and autumn [4,12]. However, the amount of PRG alkaloid consumed by individual sheep was not measured due to the grazing nature of the studies. Ambient temperature was not reported in one study [4], however, the other study reported ambient temperatures were within the 30–38 °C range during the days when physiological observations were recorded [13]. The current work has been able to determine the hyperthermic effects of perennial ryegrass toxins under known ambient temperature conditions similar to those experienced during an Australian summer and autumn period, when toxin intake was known.

Leg and back skin temperature increased due to alkaloid consumption and heat. This may have been due to vasoconstriction occurring in the extremities [3] of the sheep and/or the 38 °C heat. The response from sheep was more severe in this study as compared to a previous study by the same authors which used 33 °C heat and a higher alkaloid dose [14], suggesting the higher 38 °C conditions were causative of these effects.

## 5. Conclusions

This study demonstrated that feeding alkaloids under extreme heat conditions can compromise sheep productivity in the form of decreased feed intake and may reduce sheep liveweight over a longer period of time. Alkaloid intake, when fed via seed, combined with 38 °C heat resulted in respiration rate increasing to severe levels. This is the first study to elucidate the severity of PRGT heat stress under ambient temperature conditions similar to those experienced during a severe southern Australian summer, when toxin intake was known. Interestingly, the findings also indicate that alkaloid ingestion and 38 °C heat produce a similar heat stress response. Overall, the findings suggest that producer profitability along with sheep health and welfare may be compromised when sheep are consuming alkaloids or during severe weather events, such as extreme heat, and this effect is most severe when alkaloid consumption occurs under hot conditions. Further study investigating longer term effects of alkaloids under natural southern Australian summer conditions, when toxin intake is known, is warranted to further elucidate the effects of alkaloid consumption and heat on animal production and heat stress, reflecting that experienced in the field.

## Figures and Tables

**Table 1:** Overall average daily gain (ADG), faecal water (%) and dry matter digestibility (DMD) for each treatment.

Treatment/Temperature	NilAlk		Alk		Sed ^1^	*p* Value		
	TN	Heat	TN	Heat		Alk ^2^	Temp ^3^	AlkxTemp
ADG (grams/day)	83	83	34	−34	61	0.07	0.44	0.44
Faecal water (%)	59	58	62	57	2.9	0.64	0.15	0.31
DMD (%)	57	55	58	58	2.2	0.17	0.58	0.46

**^1^** Standard error of the difference; **^2^** NilAlk *vs.* Alk; **^3^** Temperature (TN *vs.* Heat).

**Table 2:** Rectal temperature, respiration rate, back skin temperature and leg skin temperature averaged over week 1 and 2 of the experimental period for each treatment.

Treatment/Temperature	NilAlk		Alk		Sed ^1^	*p* Value		
	TN	Heat	TN	Heat		Alk ^2^	Temp ^3^	Week
Rectal temperature (°C)								
W1	39.55	39.95	40.12	40.33	0.11	<0.001	0.002	<0.001
W2	39.56	39.88	39.95	40.11				
Respiration rate (breaths/min)								
W1	77	132	135	174	11.5	<0.001	<0.001	0.77
W2	77	134	119	184				
Back skin temperature (°C)								
W1	38.40	39.05	38.82	39.31	0.13	0.004	<0.001	0.002
W2	38.32	39.01	38.63	39.17				
Leg skin temperature (°C)								
W1	38.19	38.98	38.66	39.18	0.12	0.008	<0.001	<0.001
W2	38.09	38.87	38.27	38.95				

**^1^** Standard error of the difference; **^2^** NilAlk *vs.* Alk; **^3^** Temperaure (TN *vs.* Heat).

## Abbreviations

The following abbreviations are used in this manuscript:

PRG	Perennial ryegrass
PRGT	Perennial ryegrass toxicosis
DM	Dry matter
TN	Thermoneutral
ADG	Average daily gain
DMD	Dry matter digestibility
